# Heart rate variability analysis for the identification of the preictal interval in patients with drug-resistant epilepsy

**DOI:** 10.1038/s41598-021-85350-y

**Published:** 2021-03-16

**Authors:** Adriana Leal, Mauro F. Pinto, Fábio Lopes, Anna M. Bianchi, Jorge Henriques, Maria G. Ruano, Paulo de Carvalho, António Dourado, César A. Teixeira

**Affiliations:** 1 University of Coimbra, Centre for Informatics and Systems of the University of Coimbra, Department of Informatics Engineering, Coimbra, Portugal; 2 Politecnico di Milano, Department of Electronics, Information and Bioengineering, Milan, Italy; 3 University of Algarve, Department of Electronics and Informatics Engineering, Faculty of Science and Technology, Faro, Portugal

**Keywords:** Epilepsy, Predictive markers

## Abstract

Electrocardiogram (ECG) recordings, lasting hours before epileptic seizures, have been studied in the search for evidence of the existence of a preictal interval that follows a normal ECG trace and precedes the seizure’s clinical manifestation. The preictal interval has not yet been clinically parametrized. Furthermore, the duration of this interval varies for seizures both among patients and from the same patient. In this study, we performed a heart rate variability (HRV) analysis to investigate the discriminative power of the features of HRV in the identification of the preictal interval. HRV information extracted from the linear time and frequency domains as well as from nonlinear dynamics were analysed. We inspected data from 238 temporal lobe seizures recorded from 41 patients with drug-resistant epilepsy from the EPILEPSIAE database. Unsupervised methods were applied to the HRV feature dataset, thus leading to a new perspective in preictal interval characterization. Distinguishable preictal behaviour was exhibited by 41% of the seizures and 90% of the patients. Half of the preictal intervals were identified in the 40 min before seizure onset. The results demonstrate the potential of applying clustering methods to HRV features to deepen the current understanding of the preictal state.

## Introduction

Epileptic patients suffering from drug-resistant (or medically intractable) epilepsy (DRE) have their daily lives disrupted by the occurrence of sudden seizures. These patients, representing 30% of epileptic patients, do not benefit from anti-epileptic drug delivery^1–3^, encouraging the development of seizure-controlling methodologies. A solution involving the integration of seizure prediction models into a warning device could allow for the minimization of possible injuries and anxiety levels resulting from the unpredictability of epileptic seizures^2–4^. Envisioning such a solution, several studies have presented seizure prediction approaches designed to capture preictal electroencephalogram (EEG) patterns reflecting the transition from the normal brain state (interictal) to a state of hypersynchronous neural activity (ictal)^5,6^. Typically, most of the published seizure prediction approaches are based on supervised learning techniques that require the presence of labels for each epoch: interictal, preictal and ictal. Hence, the correct estimation of these brain states may impact the design of seizure prediction models.

However, the effective estimation of the preictal interval is still an open question. Although there is clinical evidence of the existence of this interval, it has not yet been associated with any recurrent pattern, and no consensus has been reached on its clinical definition^2–4,7^. Although no widely accepted evidence of the preictal interval has been presented to date, widespread confidence in its existence is suggested by the predictability of seizures^3,6–8^. In most of the published EEG-based seizure prediction studies, the lack of clinical knowledge has been overcome by using a fixed preictal interval, typically ranging from 2 to 90 min^2^. Additionally, a few studies have compared the performance of models for different discrete preictal intervals, ranging from 2 to 240 min^9–17^. In most of these studies, the preictal interval leading to the best performances ranged from 28 to 45 min (on average for all patients)^10–14^. However, some authors have reported optimal performance using the maximum value of the preictal interval considered (40 or 60 min)^11,13,15^. Such an outcome might suggest that this brain state may last for more than one hour. Clear differences in the durations of the preictal interval among different patients and even from seizure to seizure for the same patient have also been reported, therefore suggesting the existence of a seizure-specific preictal profile^10,11,13^.

Briefly, the supervised prediction methodologies developed to date perform an “empirical search” by testing different integer preictal durations and then selecting the duration that corresponds to the best model performance. This approach is highly dependent on accurate labelling. Consequently, it is becoming evident that the correct estimation of the preictal period (location and duration before seizure onset) may lead to enormous benefits in the development of supervised seizure prediction algorithms, as more accurate data labelling can be used in the training phase^3,18^. Given this, unsupervised methodologies may provide a significant contribution to the characterization of the preictal interval, potentially addressing the preictal variability seen among patients and among seizures in the same patient.

Additionally, non-neurological preictal alterations have also been reported in the literature. In fact, epileptic seizures have implicit manifestations in other body functions in addition to the explicit brain manifestations captured by EEG. The occurrence of these events is often associated with dysfunction of the autonomic nervous system (ANS), which is reflected in the output of both the parasympathetic and sympathetic system responses to cardiorespiratory function. Given the anatomic proximity of the ANS structures to the temporal lobe, cardiac parameters such as heart rate (HR) and heart rate variability (HRV) have been reported to capture heart rhythm oscillations that are associated with epileptic discharges typically occurring in patients with temporal lobe epilepsy^19^. The emergence of such extracerebral alterations across the pre-, post- and ictal periods^20–23^, concurrent with EEG profile changes, has prompted the acquisition of other biosignals, namely, electrocardiograms (ECGs), for performing seizure prediction^24^. This growing interest by the scientific community can be explained by the advent of wearable devices that allow the continuous acquisition of physiological signals in a more comfortable and user-friendly mode for the patient that does not require the preparation of a cumbersome EEG setup and minimizes discomfort during long-term monitoring^23,24^. In the more than 30 years of research on HR and HRV changes before and during epileptic events, a majority of studies have compared HR and HRV parameters obtained for discriminating healthy controls and epileptic patients or interictal vs ictal intervals. Additionally, HRV measurements have been used as the standard parameter when studying cardiac autonomic control^8^. More recently, great interest has been expressed in HRV modulation across interictal and preictal intervals. Two studies documented HRV differences between seizure-free periods and up until 30 min of assumed preictal activity^25,26^. Four recent HRV-based studies reported promising results regarding the feasibility of seizure prediction using HRV features^27–30^. These comprehensive studies reported fixed preictal intervals of a maximum of 5 and 15 min and achieved similar results.

Based on the above, the present study was designed to provide a deeper understanding of the preictal period using easy-to-record information from HRV. First, we extracted an HRV-feature dataset from 5-min windows located in the 240 min before seizure onset^13,25^. Second, we applied clustering methodologies to all three-feature combinations from the extracted 32 HRV features to identify and characterize a seizure-specific preictal interval in the 120 min preceding seizure.

## Methods

### Study assumptions

This study was conducted assuming that (i) seizures separated by at least 240 min were considered independent events; (ii) although 240 min of data were analysed, only the cardiac changes observed within the 120 min before the seizure discharge were influenced by that event, with the data in the 240–120-min interval before seizure onset considered the minimum data required to represent interictal state; (iii) the search for the preictal interval was undertaken for solutions comprising two clusters; (iv) given the higher probability of a preictal interval lasting less than an interictal interval, the smaller cluster found in each two-cluster solution represented the preictal interval; and (v) this interval may not occur strictly near the seizure onset but could be captured as an ECG-related event eventually preceding an EEG seizure onset. A visual representation of these assumptions is depicted in Fig. S1 in Supplementary Section 1 online.

### Database

The dataset used in this study was selected from the European Epilepsy Database, also known as the EPILEPSIAE database (www.epilepsy-database.eu) and built by the FP7 EPILEPSIAE project (www.epilepsiae.eu). The database contains long-term and simultaneous EEG and ECG recordings of DRE patients undergoing pre-surgical monitoring at the epilepsy centres of Epilepsiezentrum, Universitätsklinikum Freiburg (Germany), Centro Hospitalar e Universitário de Coimbra (Portugal), and Hôpital de la Pitié-Salpêtrière, Paris (France)^31,32^. The dataset also contains a vast amount of information regarding patient etiologies and medication and seizure characteristics (including classification, lobe localization, vigilance state, EEG and clinical onset and sleep quality). Data acquisition and consequent research use were approved by the local ethics committees of the three hospitals involved in the database development (Ethik-Kommission der Albert-Ludwigs-Universität Freiburg; Comité consultatif sur le traitement de l’information en matière de recherche dans le domaine de la santé, Hôpital de la Pitié-Salpêtrière; and Comité de Ética do Centro Hospitalar e Universitário de Coimbra). Informed consent was obtained from patients and the parents and/or legal guardians of patients under 18 years of age. All methods were performed following the relevant guidelines and regulations.

From the EPILEPSIAE database, a group of patients with temporal lobe epilepsy (TLE), was selected for analysis in the present study (details for each patient can be found in Supplementary Section 2 online). This choice was based on three facts: (i) TLE is the most frequent type of focal epilepsy in adults^33^; (ii) the temporal lobe is the predominant lobe of seizure onset in EPILEPSIAE; and (iii) disturbances in the ANS manifest predominantly in patients suffering from seizures originating from the temporal lobe. Most of the structures responsible for autonomic cardiovascular regulation are localized to the same cranial region^23^. Additionally, the dataset comprises data from 41 DRE patients (24 males; age range: 13-67 years; mean age: $41\pm 16$ years) collected at the Epilepsiezentrum, Universitätsklinikum Freiburg. ECG data were acquired at a sampling frequency of 256 Hz.

To investigate the existence of a preictal period before seizure onset, only the four hours (240 min) preceding the seizure event were analysed. In this way, 150 seizures separated by less than 240 min were discarded from a total of 388 seizures, leading to the 238 seizures considered in this study.

### Extracting HRV from the ECG

**Figure 1:**
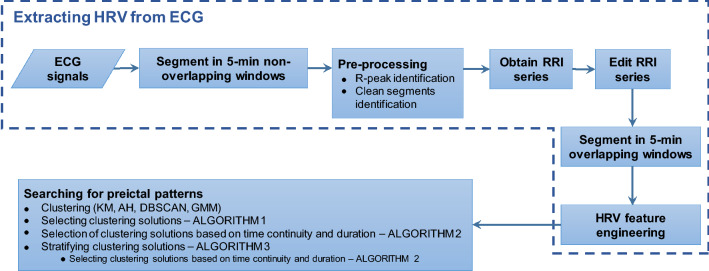
Block diagram of the proposed methodology.

Figure 1 presents the flow diagram of the proposed methodology. First, the ECG was inspected by identifying the R-peaks and clean segments. Afterwards, the intervals between subsequent R-peaks (or RRIs) were obtained, yielding the RRI series. The latter was then edited by identifying and correcting abnormal RRIs. The last step consisted of computing the HRV features. Each aforementioned step is thoroughly described in Supplementary Sections 3 and 4 online.

Linear time- and frequency-domain and nonlinear measures were extracted from 5-min ECG windows (see Table 1)^34–36^. Supplementary Section 4 online includes additional details on how each feature was obtained. From the feature engineering step (see Fig. 1), a three-dimensional matrix, $M\in \mathbb {R}^3$, with dimensions $F\times S\times W$ was obtained, with $f = 1:F, \quad F = 32$ features; $s = 1:S, \quad S = 238$ seizures; $w = 1:W, \quad W = 2768$ 5-min 98.33% overlapping windows.

**Table 1:** HRV-derived features.

inearity/Domain	Features
Linear/Time domain	NN50, pNN50, SDNN, RMSSD, SDSD, RRMean, RRMin, RRMax, RRVar
Linear/Frequency domain	Total power, VLF power, LF power, HF power, LF norm, HF norm, LF/HF
Nonlinear	SD$_1$, SD$_2$, SD$_1$/SD$_2$, DFA $\alpha _1$, DFA $\alpha _2$, ApEn, SampEn, LLE, CD
	RQA (REC, L, TT, DET, LAM, ENT, L$_{max}$)

### Searching for preictal patterns using unsupervised learning

The existence of a preictal interval, characterized by changes in HRV in the two-hour period before seizure onset, was investigated using unsupervised learning. The screening of these 120-min intervals was performed according to the indicators from the literature addressed in the Introduction. In addition to the preictal interval duration, the present study also aimed to elucidate the localization of this interval. In other words, we hypothesize that the preictal interval might not manifest for all features at the same time but rather at different timestamps for distinct groups of features. Additionally, assuming the existence of a preictal brain state in different time windows for different groups of features, this state may only manifest over an interval separated from the seizure onset instead of strictly near the seizure event. This means that a cerebral trigger might be issued as an indication of the transition from the normal brain state (interictal) to an “abnormal, hypersynchronous ictal” state^5^, which in turn can induce an abnormal state in the ANS. This trigger may be responsible for a short-term alteration in the feature values and may be expressed minutes to hours before seizure onset. Additionally, when translating this knowledge to the implementation of seizure prediction models, we might ideally expect the preictal interval to be located in the time preceding the seizure prediction horizon (SPH), a minimum interval separating alarm onset from seizure onset. Given the potential to integrate the preictal interval in the seizure prediction methodology to allow for the patient or the caregiver to prepare for an upcoming seizure, optimally, the preictal interval should be found before this SPH interval^37^. In this work, an SPH of 10 min was considered suitable for realistic application.

The aforementioned hypotheses are reflected in the analysis of the results obtained after performing clustering on the feature dataset, as described hereafter. The clustering task was conducted for all three-feature combinations from among the $F=32$ feature dataset:

- $c = 1:C, \quad C = 4960$ combinations of three features resulting from $C^F_3$.

In this way, it might be possible to understand which features are more frequently present among the best clustering solutions and therefore, by preserving the original semantics of the feature dataset, provide a simple interpretation of the clustering results. Additionally, we maximized the probability of discovering interesting solutions by combining features three-by-three instead of only examining the two-dimensional feature space.

The following clustering methods were applied to each of the 4960 three-dimensional feature spaces:

1. K-means clustering (KM), a partitioning method typically successful in detecting spherically shaped and well-separated clusters.
2. Agglomerative hierarchical clustering (AH) is often used to identify structured clusters. Here, the distance between clusters was measured using the Ward method and the Euclidean distance metric^38^.
3. Density-based spatial clustering of applications with noise (DBSCAN) is considered appropriate for identifying structured clusters while distinguishing noisy samples or outliers. Two parameters should be defined: the minimum number of samples in clusters, *MinPts*, and a radius Euclidean distance, $\varepsilon$, that allows establishment of a neighbourhood among samples^39,40^. Here, *MinPts* was set to six, which is twice the dimensionality of the feature space according to Sander *et al.*^41^. Four different values of $\varepsilon$ were tested after data normalization and analysis of the k-distance plot (resulting in DBSCAN$_\varepsilon$, with $\varepsilon =$ 1, 2, 3 and 4)^39^.
4. Expectation-maximization (EM) clustering using Gaussian mixture models (GMMs)^40^, applied by assuming clusters follow a Gaussian distribution and are therefore described by a mean and standard deviation (both parameters estimated using the EM algorithm).

#### Selecting clustering solutions

**Figure 2:**
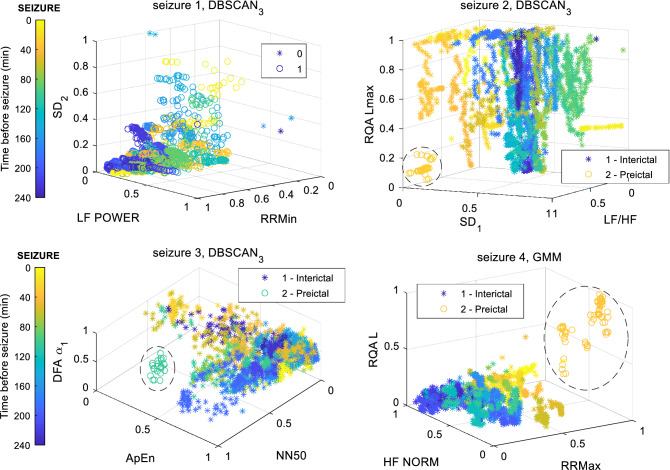
Representation of clustering solutions for patient 5. The smaller clusters (identified with dashed lines) found for the second and fourth seizures are continuous, lasting for 3.50 and 5.42 min (or comprising 43 and 66 samples), respectively. The smaller cluster for the third seizure is discontinuous and lasts for 1.83 min (i.e., 23 samples). The three accepted solutions (for the last three seizures) correspond to Dunn’s index (DI) values of 0.1555, 0.1576 and 0.1585, respectively. No clustering solutions were accepted for the first seizure; a solution containing noisy samples was randomly selected and is represented here.

We assume that the preictal interval can be represented by a single cluster, clearly separated from the remaining samples. This is the reason why we (i) defined $k = 2$ for the KM, AH and GMM methods and (ii) searched for DBSCAN clustering solutions with two clusters. Additionally, solutions containing noisy samples, sometimes returned by DBSCAN, were discarded from the analysis. When the assumed preictal interval, corresponding to the smaller of the two clusters with the lowest number of samples (see Fig. 2), was found to contain less than 1.58 min of information (less than 20 samples), it was also considered noise and therefore excluded from the results^17^. The clustering solutions previously obtained were then evaluated using Dunn’s index (DI)^40^. Clustering solutions representing compact and well-separated clusters were characterized by high DI values. A minimum DI value was defined to accept a given clustering solution^42^. Specifically, if a solution presented a DI equal to or above 0.15 (defined according to Mahallati et al.^42^ and by visual inspection of solutions across all patients), then it was assumed to identify a preictal interval. With this strategy, the initial set of 4,960 clustering solutions, inspected for each seizure and clustering method, was drastically reduced by considering the aforementioned criteria for accepting solutions (see algorithm 1 in Supplementary Section 5 online). Specifically, only 0.92% of the solutions were selected in this step.

#### Selecting clustering solutions based on time continuity and duration

Given that different feature combinations and different clustering methods could yield more than one clustering solution, the solutions selected in the previous step were differentiated by using two indicators: time continuity and duration (i.e., number of samples). The preictal interval was then classified (see algorithm 2 in Supplementary Section 5 online) as continuous if the samples in the smaller cluster were sequential over time and discontinuous otherwise.

The first criterion, time continuity, was considered by reasoning that a given clustering solution represented a preictal interval occurring continuously over time. If no time continuity was observed for the smaller cluster, it might indicate the existence of “jumps” from a preictal interval to an interictal state evolving towards seizure onset. In addition, solutions containing a continuous smaller cluster were selected over discontinuous clusters since a continuous preictal interval meant that a clearer and permanent change occurred before seizure onset.

When more than one solution was found (after selection by time continuity) and when those solutions comprised smaller clusters of different sizes, the solution for which the smaller cluster had the highest number of samples was chosen, as it provided more statistical confidence in the presence of a preictal state.

#### Stratifying and selecting clustering solutions based on time continuity and duration

Finally, another analysis was performed to provide quantitative information regarding the location of the assumed preictal interval (see algorithm 3 in Supplementary Section 5 online). To this end, the temporal location of the samples defining the smaller cluster was registered and stratified into the following intervals: 120 to 80 min, 80 to 40 min and 40 to 0 min before seizure onset. This analysis enabled an understanding of how many clustering solutions comprise a smaller cluster (assumed as the preictal interval) starting in one of the three 40-min intervals near the seizure.

The MATLAB source code developed for this study is publicly available on GitHub via https://github.com/adrianaleal/HRV-Preictal-Identification-Epilepsy.git.

## Results

Figure 2 depicts an example of the clustering solutions returned for patient 5. Evidence of a preictal interval was found for three of the four seizures; the interval was continuous over time for the second and fourth seizures. For this patient’s first seizure, it was not possible to find clustering solutions complying with the conditions of the first selection process. In other words, for this seizure, there were no clustering solutions comprising a smaller cluster with a minimum of 20 non-noisy samples (or lasting for at least 1.58 min) and a cluster validity index, in this case DI, over 0.15.

### Selecting clustering solutions based on time continuity and duration

**Figure 3:**
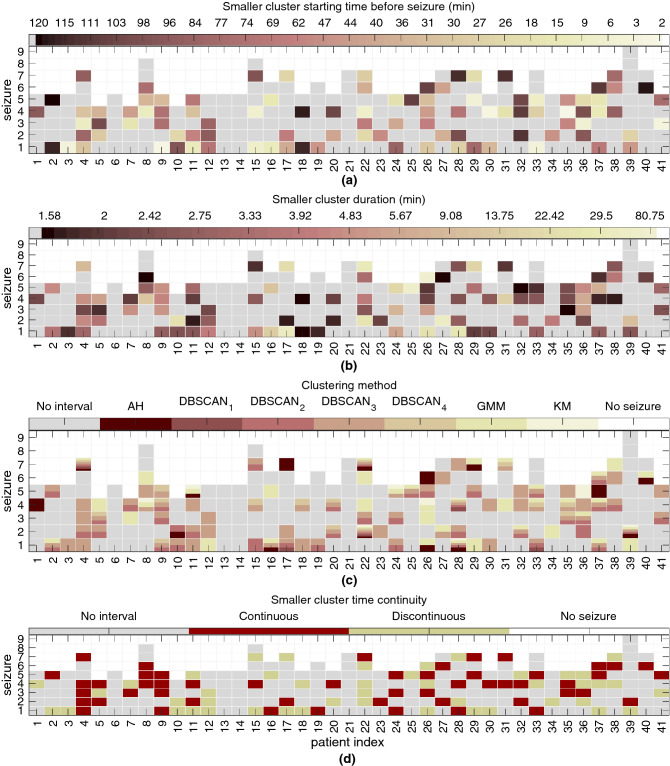
Results for the selection of clustering solutions based on time continuity and duration. The smaller cluster from the selected two-cluster solutions found for 97 seizures was characterized. The colours in the figure indicate (**a**) the smaller cluster start time before seizure onset (0 min); (**b**) the duration of the smaller cluster; (**c**) the clustering methods returning the clustering solutions; and (**d**) the continuity over time of the smaller cluster (54% were continuous clustering solutions). See the colour scale for each subfigure. The x-axis and y-axis in all plots contains the patient and seizure indexes, respectively. As an example, the clustering solution accepted for seizure 1 of patient 24 was returned by DBSCAN$_2$ and DBSCAN$_3$ and comprises a continuous smaller cluster that starts 84 min before seizure onset and lasts for 5.67 min.

In Fig. 3, we analyse the clustering results obtained by selecting solutions according to time continuity and duration for the smaller cluster. Solutions were accepted for a total of 97 seizures out of 238 (41%). Additionally, 35 of the represented solutions contain a smaller cluster that occurred or just ended in the SPH interval of 10 min before seizure onset. In other words, an assumed preictal interval was found before the SPH interval for 62 seizures (26%).

According to Fig. 3, the smaller cluster can be characterized in terms of the start time before seizure onset, duration, clustering methods used in its generation and time continuity. In terms of time continuity, there were 52 solutions that were continuous over time (54%). Additionally, the continuous smaller clusters were found to last from 1.58 min (20 samples) to 35.83 min (431 samples), whereas the duration of discontinuous clusters usually fell in the range of 1.58 min (20 samples) from 80.75 min (970 samples). Among the clustering methods, DBSCAN$_3$, DBSCAN$_2$ and GMM returned the vast majority of accepted clustering solutions (34%, 22% and 19%, respectively). Finally, the start time of the smaller cluster demonstrates high variability. Therefore, to better quantify the start time of the smaller clusters, we performed stratification of the solutions into three intervals and present the results in the following section.

### Stratifying and selecting clustering solutions based on time continuity and duration

**Figure 4:**
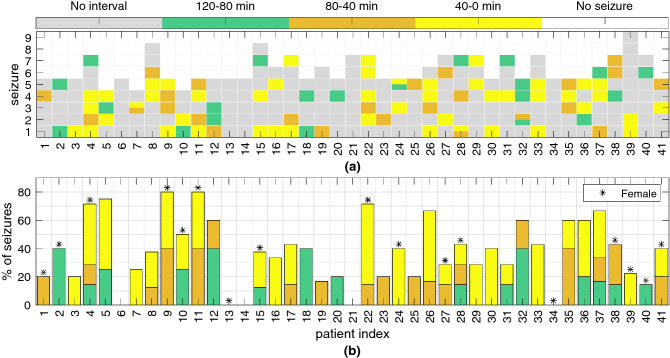
Results of the stratification and selection of clustering solutions based on time continuity and duration. (**a**) Information regarding the existence of clustering solutions for each patient (x-axis) and each seizure (y-axis, in chronological order) for the three different intervals considered: 120–80, 80–40 and 40–0 min before seizure onset (occurring at 0 min). There were four seizures (third seizure in patient 7, fifth seizure in patient 24, first seizure in patient 28, and second seizure in patient 32) for which clustering solutions were found in more than one interval. (**b**) The percentage of seizures for which at least one clustering solution was found is depicted for each patient and each 40-min interval considered. Female patients are indicated by an asterisk. It is important to note that when clustering solutions were found in more than one interval, the interval nearer the seizure was considered for the computation of the percentage for each patient in this subfigure.

The clustering results were subsequently stratified into three 40-min-long intervals occurring before seizure onset (120–80, 80–40 and 40–0). The results, presented in Fig. 4, indicate that for 89 seizures out of 238 (37%), it was possible to find clustering solutions comprising a smaller cluster suggestive of the existence of a preictal interval. In fact, there were 15 solutions found in the previous subsection that could not be stratified into the intervals considered. However, for seven of those seizures, other accepted solutions were found to fit in those intervals. These solutions contained a smaller cluster that was discontinuous and/or had a shorter duration than the solution selected in the previous subsection. For the remaining eight seizures, no clustering solution fit in the 40-min intervals.

Whereas no clustering solutions were found for any of the seizures from four patients, there were 12 patients for whom it was possible to determine solutions for 50% or more of the seizures. Additionally, 40–0-min intervals were more prominent (found for 47 seizures, 53%) than the other two intervals (120–80-min intervals found for 21 seizures, 28%, and 80–40-min intervals found for 25 seizures, 24%).

The results were cross-checked with metadata provided by the EPILEPSIAE database, including the four variables characterizing each patient: sex (male and female), epileptic focus lateralization (right, left and both hemispheres), age at hospital admission and age at the time of the first epileptic seizure (onset age). However, no correlation was found between our results and these variables, which, for the case of lateralization, is in line with the literature^22,23^.

**Figure 5:**
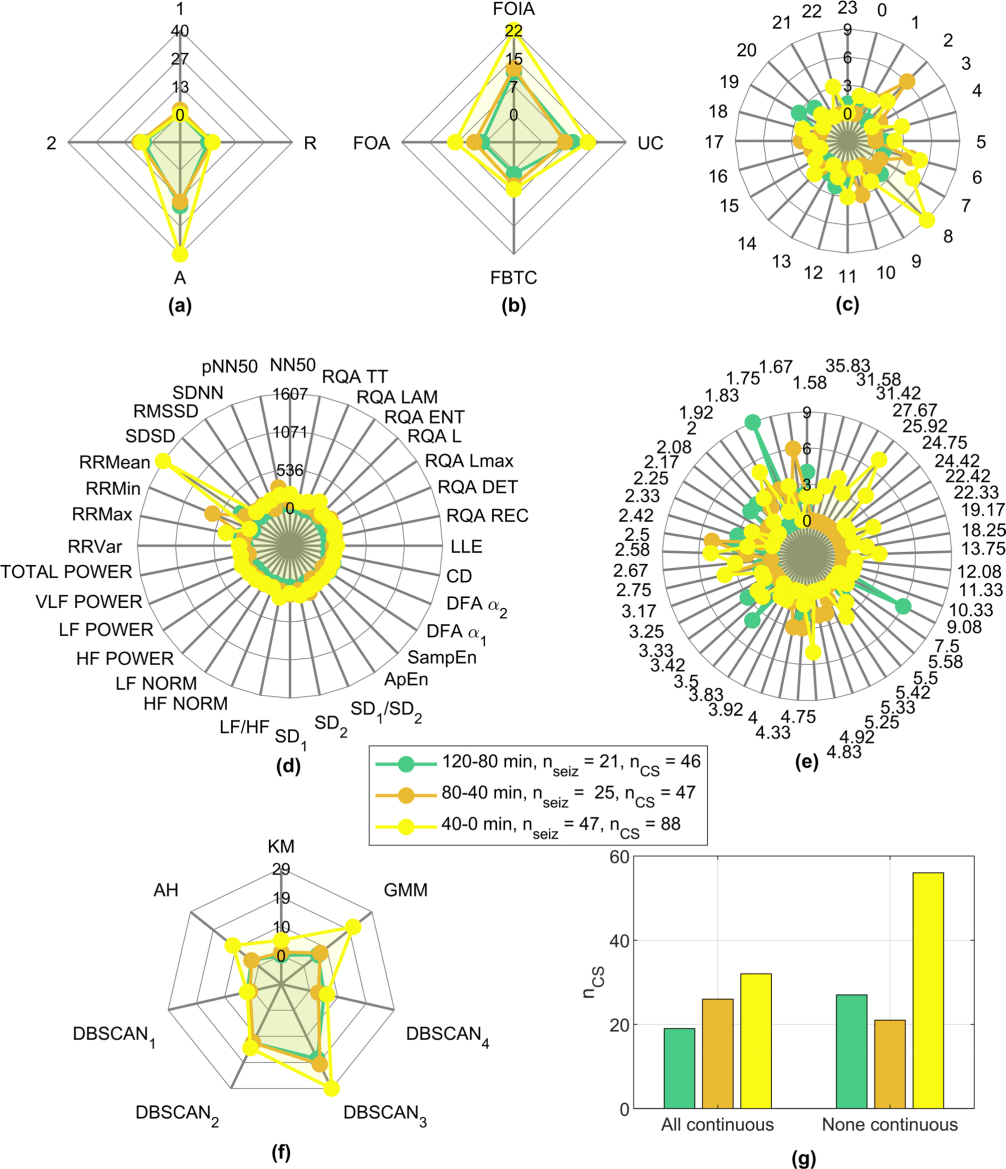
Characterization of the clustering results for each 40-min interval. (**a**) Seizure vigilance state (A: awake, 1: sleep stage I, 2: sleep stage II, R: REM sleep), (**b**) seizure type (FOA: focal onset aware, FOIA: focal onset impaired awareness, FBTC: focal to bilateral tonic-clonic, UC: unclassified), (**c**) seizure onset time, (**d**) most frequent features, (**e**) duration of the smaller cluster (minutes), (**f**) most frequent clustering methods and (**g**) time continuity of the smaller cluster (“All continuous” was assigned when all solutions found for that seizure were continuous and “None continuous” if no solution was continuous). $n_{CS}$ indicates the number of clustering solutions found in each interval, including when more than one solution was found for each seizure.

The metadata characterizing each seizure were also analysed (see Fig. 5a–c). Seizures that occurred in the awake stage as well as focal onset impaired awareness (FOIA) seizures were predominant among the accepted clustering solutions. However, seizures occurring when the patient was awake were the most frequent among all 238 seizures (76%). The same occurred with FOIA seizures, which were the most frequent type of seizure (50%). When interpreting the results according to the seizure onset time, no strong conclusion could be drawn, apart from a slight tendency for the seizures to occur early in the morning among those clustering solutions that were accepted, specifically for the 40–0-min interval before seizure.

The smaller clusters observed among the two-cluster non-noisy solutions (assumed to represent preictal intervals) were further characterized in terms of the most frequent features and clustering methods, as shown in Fig. 5d,f, respectively. All the obtained solutions were also analysed in terms of the duration and continuity of the smaller cluster (see Fig. 5e,g, respectively). Here, it is important to note that the numbers on the axis for (d–g) do not add up to the number of seizures for which several clustering solutions were accepted (as occurs in (a–c)). In fact, for the same seizure, it was possible to find clustering solutions complying with the preictal interval requirements for different (i) 40-min intervals (see patients 7, 24, 28 and 32 in Fig. 4), (ii) clustering methods and (iii) feature combinations.

Regarding the features most frequently appearing in the accepted clustering solutions, it is clear that time-domain features such as RRMax, RRMin and RRMean are strongly predominant. Additionally, LF/HF, pNN50, NN50 and RQA ENT also stand out. It is also worth noting the differences between the profile presented for 40–0 min intervals and that for the other two 40-min intervals, as clearly manifested, for example, in features RRMean, RRMin, RRMax and pNN50. Such differences might indicate the occurrence of HRV changes over time that are captured by different groups of features in distinct time intervals. The fact that the mean, minimum and maximum of the RR intervals were often observed among the accepted clustering solutions may indicate the presence of ECG confounds that are not related to the occurrence of epileptic seizures.

Concerning the tested clustering methods, it can be seen that methods GMM and DBSCAN (applied with $\varepsilon = 2$ and $\varepsilon = 3$) returned the majority of accepted solutions, therefore indicating that these methods are most suitable for searching for preictal intervals among clustering solutions of diverse shape and duration.

With regard to the duration of the smaller cluster, no one cluster duration demonstrates clear prevalence, which means that when assuming a preictal interval corresponding to this smaller cluster, it is likely to have a duration that lasts from 1.58 min (20 samples) up to a maximum of 35.83 min (431 samples). It is worth noting that this limit applies for the 40–0-min interval, whereas a maximum number of samples corresponding to 9.08 min (110 samples) and 5.42 min (66 samples) was returned for 120–80- and 80–40-min intervals, respectively. It can also be observed that the duration of the clustering solutions was found to range from 1.58 to 9.08 min for 100% of the clustering solutions found for the 120–80- and 80–40-min intervals and in 58% of the solutions fitting 40-0-min intervals. These results further support the hypothesis that different seizures, possibly associated with the same patient, are characterized by different preictal dynamics^3,18^.

Finally, a larger percentage of discontinuous clustering solutions was found for the 40–0-min interval than for the other intervals. This indicates that cardiac events triggered as a result of an oncoming seizure that are located in the 40 min before the seizure most likely do not occur continuously but rather as a sequence of heart rhythm alterations occurring towards the seizure onset. It is worth mentioning that the noise detection method may introduce missing values responsible for pseudo-discontinuities.

## Discussion

This study is a proof of concept that is, to the best of our knowledge, the first attempt to apply unsupervised learning methods to HRV-derived features in characterizing the preictal interval. Evidence of this interval was found in 41% of the seizures analysed and in 37 out of 41 patients. In addition, preictal intervals ending before the seizure prediction horizon of 10 min were found for 26% of the seizures as well as a total of 54% continuous preictal intervals. Furthermore, 53% of the preictal intervals occurred in the 40 min before seizure onset, which is in line with the mean duration and location of preictal intervals leading to the best seizure prediction performances in previous studies^9–15^. For the majority of the clustering solutions, the duration of the preictal interval ranged from approximately 2 to 9 min. The results also show the high variability of this interval both between and within patients, reinforcing the need for patient-specific approaches in treating epilepsy^3,18^.

With regard to the most relevant features identified in this study, there was a clear prevalence of time-domain features such as RRMax, RRMin and RRMean along with a mild presence of LF/HF, pNN50, NN50 and RQA ENT among the accepted clustering solutions. These results are aligned with those presented in two HRV-based seizure prediction studies^28,30^. Behbahani et al.^30^ reported RRMean, LF/HF and SD$_1$/SD$_2$ as the features capturing most changes in the preictal state. They developed a seizure prediction model using an adapted decision threshold method and a preictal interval of 5 min. Billeci et al.^28^ proposed a seizure prediction model based on HRV features, using a preictal interval of 15 min and at least 50 min of interictal data. To the best of our knowledge, to date, this is the only study presenting a comprehensive analysis of an HRV feature dataset in terms of the number and importance of each of those features in distinguishing interictal from preictal epileptic stages. In fact, after applying a feature selection method, the authors found that features obtained from the time (RRMean, pNN50) and frequency (HF/LF) domains, together with nonlinear measures (RQA LAM, HF power and coefficient of SampEn), were relevant in characterizing a preictal interval of 15 min.

While identifying a preictal stage in 41% of seizures is not sufficient for developing a seizure prediction model, we did not expect to observe cardiac changes for all seizures^3,8,10^. It might not even be possible to find preictal patterns in electrographic data, as reported by Bandarabadi et al.^10^. In that study, an optimal preictal period was found for 67% of the seizures using a method based on amplitude distribution histograms and spectral features extracted from EEG data^10^. In sum, monitoring autonomic changes might prove useful in seizure prediction only for some patients or even for specific seizures recorded for the same patient^3^.

Additionally, the results reported herein should be understood in light of the limitations of our study. Namely, the assumptions regarding the search for the preictal interval, taken for the sake of finding acceptable solutions, may have made our unsupervised approach not completely unsupervised. The analysis of 240 min of ECG data may also weaken confidence in the existence of a sufficient amount of interictal cardiac screening. However, in addition to the two studies in the literature reporting the EEG and ECG analysis of this time interval^13,25^, the vast majority of supervised studies in the literature indicate that the preictal interval is located within an hour before the seizure onset^10–15^. Accordingly, we found that more than 53% of seizures manifested preictal HRV changes in the 40–0-min interval. As a result, we consider that a representative interictal interval was analysed for each seizure, simultaneously allowing a fast computation of the results. Accordingly, it is important to highlight that new studies are required to confirm the existence of such intervals in certain seizures using HRV data. In fact, new research on both cardiac and brain information can uncover the types of seizures for which pre-seizure changes are common. We also encourage new endeavours in the unsupervised characterization of the preictal interval, as we believe this new perspective has the potential to reveal key aspects related to neurophysiological knowledge of the preictal state.

Two of the previously addressed aspects establish future directions for the presented work. Specifically, the additional information regarding cardiac preictal changes could improve seizure prediction methodologies, particularly in the context of multimodal approaches. Future research will consist of applying the methodology described herein to the EEG recordings of the same group of patients to validate the results for the preictal interval found by ECG analysis. In this way, it will be possible to overcome our current main limitation, i.e., validation of the origin of the cardiac changes seen over the 240 min of data. Performing an unsupervised search of the preictal interval on EEG data could make it possible to discard potential confounders present in the ECG and EEG signals and increase confidence in the identified preictal intervals. In addition to assessing the neurological condition of the patient, an EEG analysis may also allow the identification of artefacts (e.g., muscular artefacts). This information can be used to eliminate confounding factors for the unsupervised preictal interval search in ECG. For instance, muscular artefacts may result from walking or talking and may be associated with an alteration of the heart rhythm. In sum, the characterization of the preictal interval based on EEG and ECG will yield new labels for the preictal interval, which will afterwards be integrated into data fusion and seizure-specific prediction methodologies. The final results are expected to contribute to the field of epilepsy in terms of the design of prospective seizure prediction studies, recognized in the epilepsy field as a path leading to the validation of the clinical applicability of prediction models^3^.

Ultimately, the evidence of preictal changes may enable the prediction of epileptic seizures sufficiently early to allow the patient to prepare for the upcoming seizure, seek a safe location to experience the seizure and avoid negative social exposure during seizure occurrence. Moreover, as the field of epilepsy progresses, the feasibility of seizure prediction might lead to the development of new strategies for therapeutic treatment, such as closed-loop electrical stimulation, enabling seizure control. Considering the path to such clinical applications, further studies are required to address this work’s limitations regarding the analysis of data acquired during pre-surgical monitoring. Even though we are aware of the influence of alterations of medication on the ANS and, therefore, on the ECG, we believe that the seizure-specific approach taken in this study allows a normalization of the medication effect at the individual level.

## Supplementary information

Supplementary Information
