# Supplementary material for: Heart rate variability analysis for the identification of the preictal interval in patients with drug-resistant epilepsy
Source: Sci Rep. 2021 Mar 16;11:5987. doi: 10.1038/s41598-021-85350-y (PMC7966782; doi:10.1038/s41598-021-85350-y)
Supplement: Supplementary file 1 — Supplementary Information [file 41598_2021_85350_MOESM1_ESM.pdf]

Supplementary information for:

Heart Rate Variability Analysis for the Identification of the Preictal Interval in Patients with Drug-resistant Epilepsy

Adriana Leal, Mauro F. Pinto, Fábio Lopes, Anna M. Bianchi, Jorge Henriques, Maria G. Ruano, Paulo de Carvalho, António Dourado and César A. Teixeira

1 Study assumptions

The main goal of this study is to characterize the preictal interval using unsupervised methods. In sum, we applied seven clustering methods (K-means, agglomerative hierarchical, density-based spatial clustering of applications with noise using $\varepsilon = 1$, 2, 3 and 4 and Gaussian mixture models) to all combinations of three features in the $F = 32$ feature dataset (4960 feature combinations per seizure). We obtained a total of 34720 clustering solutions per seizure that we analysed aiming at characterizing the preictal interval. Accordingly, we settled the following assumptions (represented in Fig. S1) to identify what we considered could be valid clustering solutions:

(i) Seizures separated by at least 240 minutes were considered independent events.

(ii) Although 240 minutes of data were analysed, only the cardiac changes observed within the 120 minutes

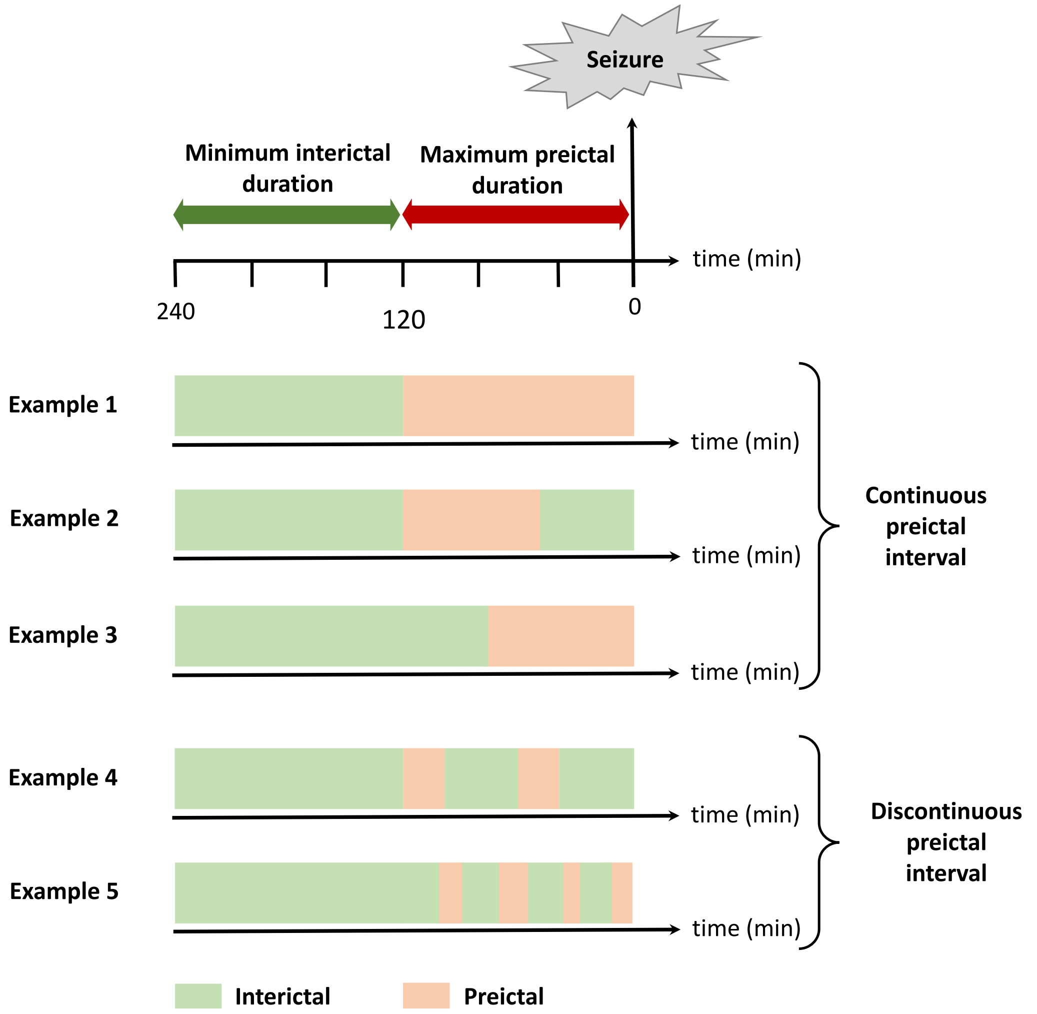

Figure S1: Examples of preictal location and duration along the 240 min of analysed HRV data.

before the seizure discharge were influenced by that event, with the data in the 240-120-minute interval before seizure onset considered the minimum data required to represent interictal data (see Fig. S1).

(iii) The search for the preictal interval was undertaken for solutions comprising two clusters.

(iv) Given the higher probability of a preictal interval lasting less than an interictal interval, the smaller cluster found in each two-cluster solution represented the preictal interval (see Fig. S1).

(v) This interval may not occur strictly near the seizure onset but could be captured as an ECG-related event eventually preceding an EEG seizure onset (see Examples 2 and 4 in Fig. S1).

2 Seizure metadata

Table S1 contains information regarding each patient, namely, sex, age at hospital admission and onset age (corresponding to the occurrence of the first epileptic event), epileptic focus lateralization and the number of seizures analysed for each patient.

In Fig. S2 it is possible to find information about the seizure classification, vigilance state and onset hour, respectively, annotated for each non-discarded subsequent seizure. Fig. S3 depicts a histogram of the number of seizures (out of a total of 238) for which the time interval between that seizure and the previous one lies in each of the intervals defined in the x axis.

Table S1: Dataset description.

#	ID	Sex	OA/AA (years)	Lat	NS	NDS	#	ID	Sex	OA/AA (years)	Lat	NS	NDS
1	402	F	10/55	L, R	5	0	22	80702	F	14/22	B	10	3
2	8902	F	23/67	L	5	0	23	81102	M	5/41	R	13	8
3	11002	M	21/41	R	8	3	24	85202	F	4/54	L	10	5
4	16202	F	43/46	L, R	8	1	25	93402	M	40/67	L	7	2
5	21902	M	44/47	L	6	2	26	93902	M	43/50	R	9	3
6	23902	M	36/36	L	5	0	27	94402	F	29/37	R	11	4
7	26102	M	15/65	L	8	4	28	95202	F	13/50	L	14	7
8	30802	M	28/28	L, R	9	1	29	96002	M	21/58	L, R	9	2
9	32702	F	33/62	L, R	6	1	30	98102	M	2/36	L	5	0
10	45402	F	13/41	L, R	5	1	31	98202	M	3/39	R	10	3
11	46702	F	13/15	R	5	0	32	101702	M	44/52	L, R	6	1
12	50802	M	2/43	L	5	0	33	102202	M	0/17	L	28	21
13	52302	F	13/61	L	7	2	34	104602	F	8/17	L	5	0
14	53402	M	0/39	L, R	8	3	35	109502	M	40/50	L, R	10	5
15	55202	F	3/17	R, B	9	1	36	110602	M	6/56	R	8	3
16	56402	M	18/47	L, R	7	1	37	112802	M	47/52	L	6	0
17	58602	M	17/32	L	22	15	38	113902	F	16/29	R	25	18
18	59102	M	17/47	R	7	2	39	114702	F	31/22	R	25	16
19	60002	M	47/55	L, R	8	2	40	114902	F	15/16	L, R	12	5
20	64702	M	3/51	R	6	1	41	123902	F	7/25	L, R	8	3
21	75202	M	10/13	R	8	1							

ID: patient identifier. Sex: female (F) or male (M). OA: onset age. AA: hospital admission age. Lat: lateralization (L: left, R: right, B: bilateral). NS: total number of seizures annotated for each patient. NDS: number of discarded seizures, obtained as a result of the analysis of the 240 min of ECG data in between seizure onsets.

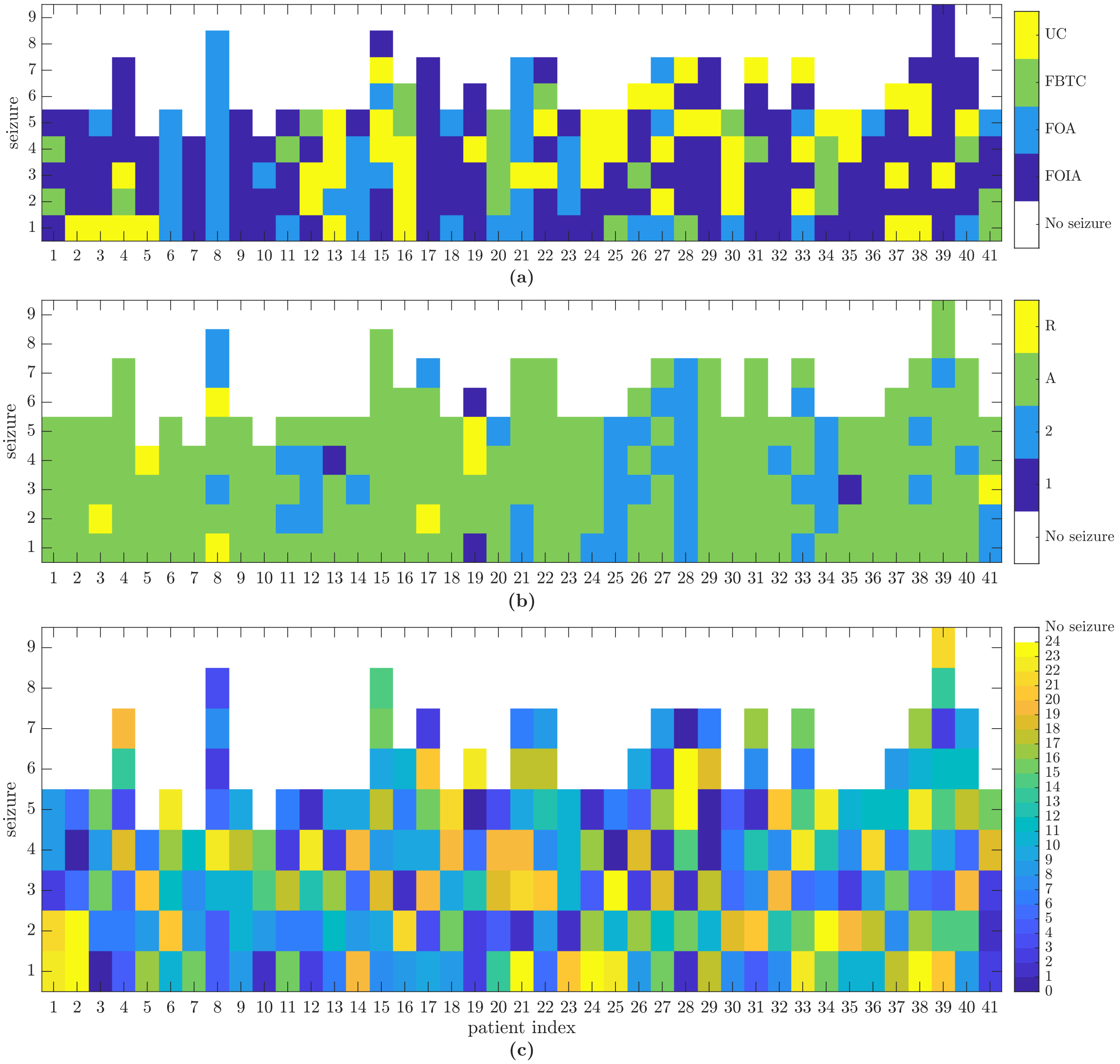

Figure S2: Information regarding each seizure in the group of patients selected for this study. (a) Seizure classification: awake (A), sleep stage I (1), sleep stage II (2), sleep stage III (3), sleep stage IV (4), REM sleep (R). (b) Seizure vigilance state: focal onset aware (FOA), focal onset impaired awareness (FOIA), focal to bilateral tonic-clonic (FBTC), unclassified (UC). (c) Seizure onset hour.

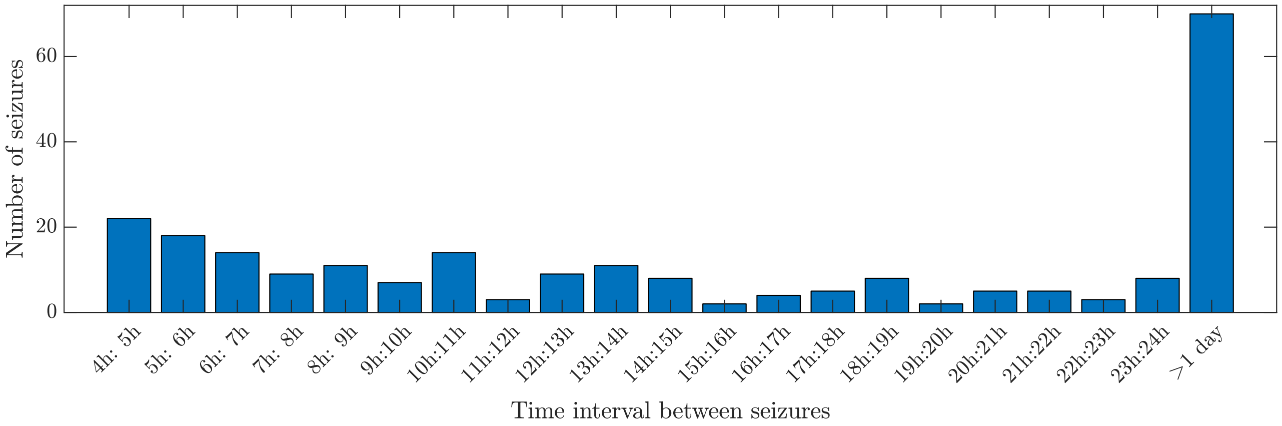

Figure S3: Histogram of the inter-seizure time intervals.

3 Extracting HRV from ECG

3.1 ECG signals pre-processing

The ECG pre-processing step (represented in Fig. S4) started by inspecting 5-min non-overlapping windows of the ECG raw data. According to the Task Force of the European Society of Cardiology and the North American Society of Pacing and Electrophysiology, five minutes is considered to be the minimum time interval required to compute HRV metrics[1]. In each 5-min window, a notch filter (IIR filter design and zero-phase distortion by forward and backward filtering) was applied at 50 Hz frequency to remove the powerline interference[5].

The next step consisted of applying the Discrete Wavelet Transform (DWT) to the 5-min non-overlapping windows, to obtain the frequencies of interest in ECG as well as to remove its baseline wander. Each window underwent a multilevel one-dimensional wavelet analysis, using a mother wavelet belonging to the biorthogonal family (*bior3.3*). This mother wavelet very closely resembles the morphology of the QRS complex[2]. As a result, ten frequency bands were obtained (ten decomposition levels and ten approximation levels). From the wavelet analysis, two frequency bands were further inspected: the approximation coefficient containing frequency content up to 45 Hz, and the approximation coefficient containing frequency components up to 0.8 Hz. The latter, comprising the baseline wander frequency components, was then subtracted from the former[3–5].

Subsequently, a R-peak detection algorithm, based on the Pan & Tompkins algorithm[6] was used to identify the R-peaks in each 5-min non-overlapping window. Similarly to Pan & Tompkins, two thresholds were computed. The first one corresponds to the application of a two-second moving average filter to the moving-window integration (using a 0.2 second window). The second one consists of a baseline threshold intended to prevent the analysis of lead-off periods. In other words, to prevent the moving average threshold from reaching zero, a baseline threshold was defined, corresponding to the minimum of the moving average threshold plus 10% of the difference between the median and the minimum of that threshold (defined experimentally). In this way, it was possible to improve the algorithm's robustness regarding the specific characteristics of the ECG of each lead and each patient.

The ECG signals were then inspected regarding the identification of noisy segments. Towards that end, a simple thresholding approach was developed, based on the computation of two measures in each 5-min window: the first detail coefficient, returned by the DWT and the zero-crossing rate. The latter was particularly useful in identifying for instance periods of lead-off signal.

3.2 RR interval series editing

The RR interval (RRI) series is obtained by computing the time intervals between adjacent QRS complexes, taking the R-peak as the reference fiducial point[7,8]. At this point, it is important to note the distinction between normal-to-normal (NN) intervals and RRIs. The former are observed between adjacent heartbeats resulting from sinus node depolarizations, whereas the latter comprises all interbeat intervals, including both the NN intervals and abnormal intervals[1]. Abnormal RRIs are obtained by analysing false beats introduced by (i) physiological artifact in the form of ectopic beats, also known as premature beats, and (ii) technical artifact originated by problems in signal acquisition, e.g., subject movement or electrode detachment, or by the

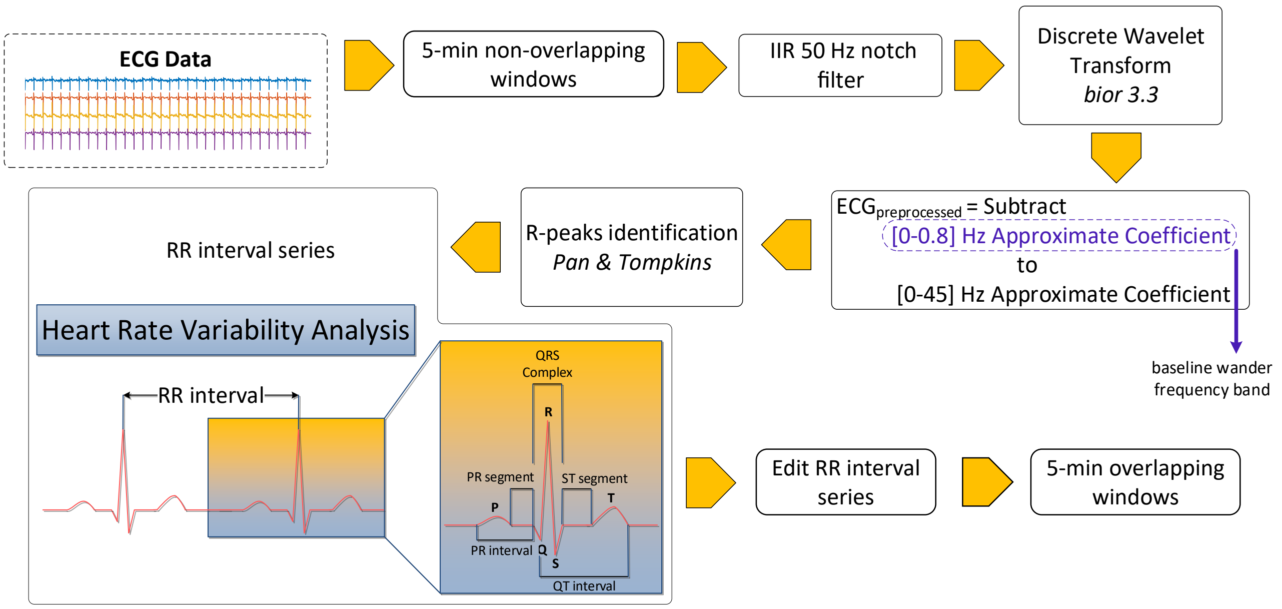

Figure S4: ECG pre-processing and RRIs series extraction.

emergence of false positives or false negatives during peak detection phase[3,7,8]. The presence of ectopic beats has been reported to undermine the HRV analysis specifically by leading to an overestimation of the values of power in the LF and HF frequency bands (described in section 4.2), and consequently of the LF to HF ratio[7,8].

To prevent the analysis of abnormal RRIs, a simple criteria has been included in this HRV analysis: if a given RRI falls outside the range of 20% around the mean of the previous ten RRIs, then either it is removed or it is replaced by an interpolated RRI value. This criterion has been built on the assumption that abrupt changes in the HR are unlikely to occur during sinus node activity[3]. When the abnormal RRI is higher than the range of values previously mentioned, it is replaced using linear interpolation[3,7,8].

From now on, the ECG signals were spanned using a 5-min (300 seconds) window with 98.33% (295 seconds) of overlapping. The RRIs corresponding to that 5-min window were pulled from RRI series, being ready for HRV-feature computation. This means that each feature sample corresponds to a time increase of five seconds. The overlap percentage was set in order to, in a future study, allow for the fusion of information from both ECG and EEG data. EEG recordings are typically analysed with a five second non-overlapping spanning window.

Furthermore, if more than 80% of the RRI series, comprised in a 5-min window, contains abnormal RRIs, that window is considered as noise and no features are extracted from it[8]. RRI and NN intervals notation will henceforth be used interchangeably to denote corrected RRIs.

4 HRV feature engineering

HRV captures the variability of the intervals between consecutive R-peaks. Such short-term oscillations reflect the neurocardiac function or, in other words, the modulation of the heart-brain interactions. In fact, an HRV analysis is typically conducted to inspect the influence of the ANS on the sinoatrial (SA) node and, this way, to assess the relative balance between the sympathetic and parasympathetic branches of the ANS (sympatho-vagal balance)[1,9]. The computation of the HRV features is intended to capture the influence of the two branches of the ANS, sympathetic and parasympathetic, responsible for maintaining homeostasis. Studies have demonstrated that, when the sympathetic nervous activity is triggered during seizures, it typically results in increased HR and blood pressure, and possible occurrence of tachycardia and tachypnea[10,11]. When the parasympathetic response predominates, for a considerably low number of seizures, the normal cardiorespiratory function is altered with regard to the decreasing of heart and respiration rates and also blood pressure[10,11].

All HRV features used in this study are presented in Table S2, with additional details regarding (i) the units of measurement; (ii) the minimum length of clean ECG signal required to compute these HRV-derived features; (iii) the number of missing values obtained for each feature resulting from the existence of noisy 5-min windows; (iv) which autonomous nervous system response has been associated with each feature (it can be both) according to the literature and (v) the computational time required to compute each feature.

4.1 HRV linear time domain features

Time domain measures of HRV are the simplest to obtain, as they are basically the result of the application of descriptive statistical methods. These measures are considered robust to a previous preprocessing of the RRI series towards artifact and/or ectopic beat removal[7]. Two types of features can be derived from the RRI series: (i) from the series of RRIs; and (ii) from the time-series resulting from the difference between successive RRIs[1].

The following time-domain features were computed:

- $NN50$: number of RRIs that last more than 50 ms.
- $pNN50$: percentage of RRIs that last more than 50 ms.
- $SDNN$: standard deviation of RRIs.
- $RMSSD$: square root of the mean squared differences of successive RRIs.
- $SDSD$: standard deviation of the differences between successive RRIs.
- $RRMean$, $RRMin$, $RRMax$ and $RRVar$: respectively, mean, minimum, maximum and variance of the RRI time-series.

SDNN corresponds to the standard deviation of the RRIs, which is equivalent to the square root of variance and, therefore, to the total power of the frequency spectrum in a given signal segment. This measure captures the cyclic components introducing variability in the ECG segment under analysis. It is important to highlight that, as the total variance of the HRV is known to increase with the length of the ECG segment, the SDNN will also be influenced by that factor. Consequently, to perform comparisons among SDNN measures, it is mandatory that the different segments analysed have the same length[1,9].

The time domain HRV features are often used as indicators of parasympathetic activity (RMSSD, NN50, pNN50 and SDSD) and overall autonomic responses (SDNN)[1,12–14].

4.2 HRV linear frequency domain features

The HRV frequency analysis was performed by computing the Lomb-Scargle periodogram[15]. This nonparametric estimation method was chosen to handle the unevenly sampled RRI series, without requiring data interpolation and, hence, do not assuming any underlying model. Using this method, it is possible to avoid the shifting of the spectral peaks towards the low frequency components and, therefore, the overestimation of the total power present in LF and HF bands[3,7].

The frequency spectrum (see Fig. S5) is inspected towards the assessment of three different spectral components: very low frequency (VLF) in the 0.0033-0.04 Hz range, low frequency (LF) corresponding to the 0.04-0.15 Hz band, and the high frequency (HF) component found in the 0.15-0.4 Hz range[1,3,7,9]. The power in LF and HF frequency bands can be normalized to the $Total\,power - VLF\,power$ entity, yielding LF norm and HF norm and thus emphasizing the influence each branch of the ANS has on the HRV regulation. Equivalently, by normalizing the frequency bands, it is possible to decrease the impact of the changes in the Total power, particularly on the values of LF and HF powers. Furthermore, it also allows for direct comparisons of power measures between two subjects. In fact, significant differences were reported between the values of Total power and power in each frequency band, among healthy subjects[1,16–20].

Eight features were then extracted from the frequency spectrum of each 5-min window:

- $Total\,power$: window's total power.
- $VLF\,power$: power in the VLF band.
- $LF\,power$: power in the LF band.
- $HF\,power$: power in the HF band.
- $LF/HF = \dfrac{LF\,power}{HF\,power}$.
- $LF\,norm = \dfrac{LF\,power}{Total\,power - VLF\,power}$.
- $HF\,norm = \dfrac{HF\,power}{Total\,power - VLF\,power}$.

Additionally, HF spectral component (or respiratory sinus arrhythmia), as the name suggests, captures HR variations associated with the respiratory cycle, therefore, reflecting the vagal or parasympathetic activity. Conversely, LF component has not yet been associated with a clear interpretation. While it is considered by the majority of the research community as an indicator of sympathetic activation, other studies suggest that LF power may be influenced by both parasympathetic and sympathetic nervous systems. As a result, the ratio of

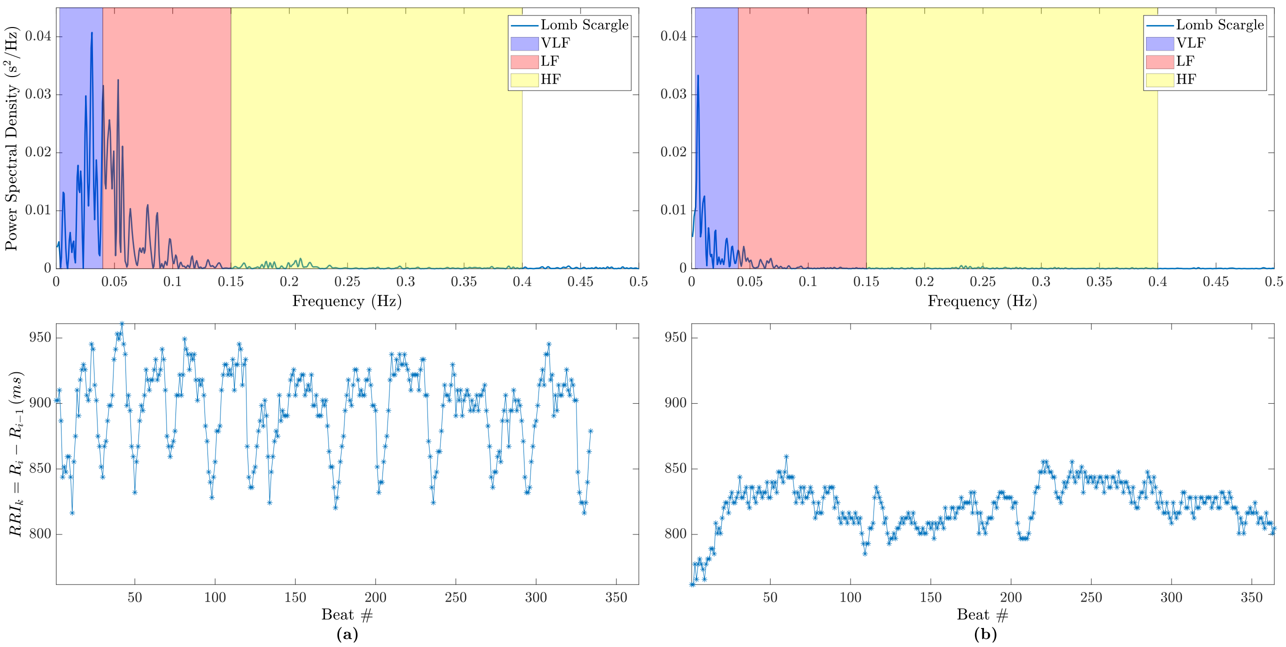

Figure S5: Examples of frequency analysis. The frequency spectrum was computed for two 5-min windows ranging from (a) 78 to 73 minutes and (b) 26 to 21 minutes before seizure onset, respectively, for the first seizure of patient 2.

LF to HF powers (typically assumed to characterize sympathovagal balance) and the VLF power remain HRV features that are not to date known to clearly manifest activity from either branch[1,10,13,14,21,22]. Finally, Total power is useful in capturing the variance of all the RRIs[14].

4.3 HRV nonlinear features

HRV is known to be the result of the complex interactions between haemodynamic, electrophysiological and humoral body functions. Additionally, the influence of the autonomic and central nervous systems' controlling actions will also be reflected in HRV metrics[1,23]. A nonlinear analysis might provide new complimentary information regarding such complex interactions. Nonlinear indices, even though not being regarded to directly capture ANS responses and, therefore, having a less clear interpretation comparing to linear measures, are known to provide higher reliability (repeatability) across measurements, when compared to linear ones[12,24]. A considerable set of nonlinear features was assembled and is described briefly below.

From the Poincaré plot representation (see Fig. S6), obtained by computing a scatter plot of each RRI against the previous one[9,25], it is possible to derive three features:

- SD_1: standard deviation of the line perpendicular to the line of identity in the Poincaré plot or, in other words, the width of the ellipse fitted to that plot.
- SD_2: standard deviation of the line of identity in the Poincaré plot, therefore measuring the length of the ellipse fitted to that plot.
- SD_1/SD_2: ratio of SD_1 to SD_2.

In other words, SD_1 and SD_2 can be regarded as measures of short- and long-term RRI variability, respectively. Accordingly, SD_1 translates parasympathetic activity, whereas SD_2 captures overall HRV[13]. Lastly, the unpredictability of the RRI series can be determined by computing SD_1/SD_2[9].

The computation of the remaining nonlinear features, which will be described hereafter, assumes that the RRI series is evenly sampled. As such, a cubic spline interpolation was performed on the irregularly sampled RRI series. This method was reported to introduce the lowest error when computing LF and HF powers[7]. An RRI time-series was obtained, characterized by a sampling frequency of 4 Hz that allows for spectral components up to 2 Hz to be captured, according to the Nyquist theorem[3,7,19].

Detrended fluctuation analysis (DFA) was performed to explore the extent of long-range correlations in the RRI time-series for different time scales and, this way, to inspect the fractal scaling properties of HRV[25–27]. Particularly, two scaling exponents were returned from DFA:

- DFA slope α_1 ($DFA\,\alpha_1$): short-term fluctuations, within the 4-11 heartbeats range.
- DFA slope α_2 ($DFA\,\alpha_2$): long-term fluctuations, over the 11-64 heartbeats range.

Two entropy measures were computed and stand as measures of regularity and complexity of a time-series:

- Approximate entropy ($ApEn$).
- Sample entropy ($SampEn$).

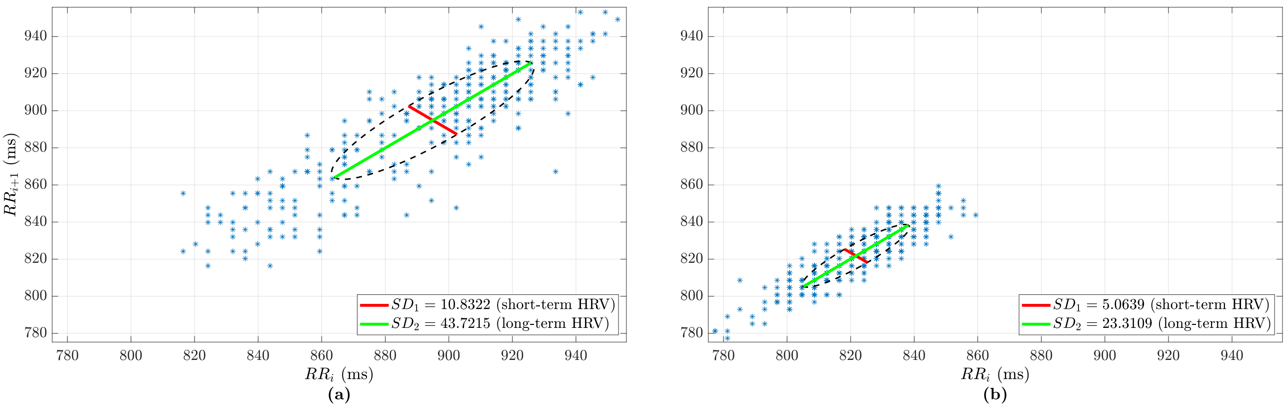

Figure S6: Examples of Poincaré plot representations. These were computed for two 5-min windows ranging from (a) 78 to 73 minutes and (b) 26 to 21 minutes before seizure onset, respectively, for the first seizure of patient 2.

By inspecting these features, it is possible to know what is the likelihood that similar sequences found for m points, within a tolerance, r, will also be found for $m+1$ points. Both measures require, therefore, the definition of the number of points, m, comprised in the sequences further compared, and the tolerance value, r, for which matches are accepted. In an HRV analysis, value of m parameter is typically set to 2. The tolerance, taken as the similarity criterion, was set to $0.2 \times SD$, SD corresponding to the standard deviation of the 5-min window of the interpolated RRI signal (of length N)[19,28–30]. A detailed description of the remaining process to compute these measures can be found in Delgado-Bonal and Marshak paper[30]. Low values of ApEn or SampEn are related to the existence of patterns, whereas high values can be found for less predictable and more complex processes[29].

By using the information of an RRI time-series to reconstruct the underlying m-dimensional dynamical system, it is possible to compute the following features:

- Correlation dimension (CD).
- Largest Lyapunov exponent (LLE).

The former quantifies the complexity of the system, whereas the latter provides information regarding the overall predictability of the system, i.e., the evolution of the trajectories in the phase space. A zero value of LLE can be found for periodic signals, whilst more chaotic systems are associated with an increase of the LLE[25,31,32]. Similarly, an increase in complexity corresponds to an increase in the value of CD[31,33]. To compute such measures, the reconstruction of the underlying system's phase space from the available measured data (RRI time-series) must be performed. In this study, the two parameters required for phase space reconstruction, the embedding dimension, m, and the time delay, τ, were estimated (for each 5-min window) using the False Nearest Neighbour algorithm and first local minimum of the average mutual information method, respectively[34–36]. The LLE was then obtained using Rosenstein *et al.* (1993)[37] method, which is one of the most widely used for this purpose, whereas CD was computed based on Grassberger & Procaccia (1983)[38] method.

A recurrence quantification analysis (RQA) is typically conducted to find hidden periodicities in the aforementioned phase space trajectory. Such information can be accessed by computing the recurrence plot (RP) of a given time-series[39]. The first step to obtain such representation is to compute the square matrix with dimensions $N \times N$ (known as colour recurrence plot and depicted in Fig. S7), containing the pairwise Euclidean distance (given by the norm $|| \cdot ||$) between all samples of the trajectory, N, in the m-dimensional space[32,40]. Afterwards, a threshold distance ε is used to define a sphere centred at the state x_i. If x_j falls within that sphere, then the Heaviside function $\Theta(\cdot)$ decides for $R_{i,j} = 1$, meaning the states are close to each other. Otherwise, $R_{i,j} = 0$. This binary matrix, symmetric along the identity line, can therefore be visualized in a black ($R_{i,j} = 1$) and white plot ($R_{i,j} = 0$), the RP. In this study, the value of ε was estimated for each 5-min window, corresponding to 10% of the maximum phase space diameter[41]. The RQA returned seven RP measures of complexity, which quantify recurrence point density and indicate the existence of diagonal and/or vertical lines in the RP. A comprehensive description of the computation of these measures can be found in[40–42]. The following RQA features were derived:

- Recurrence rate (REC).
- Determinism (DET).
- Average diagonal line length (L).

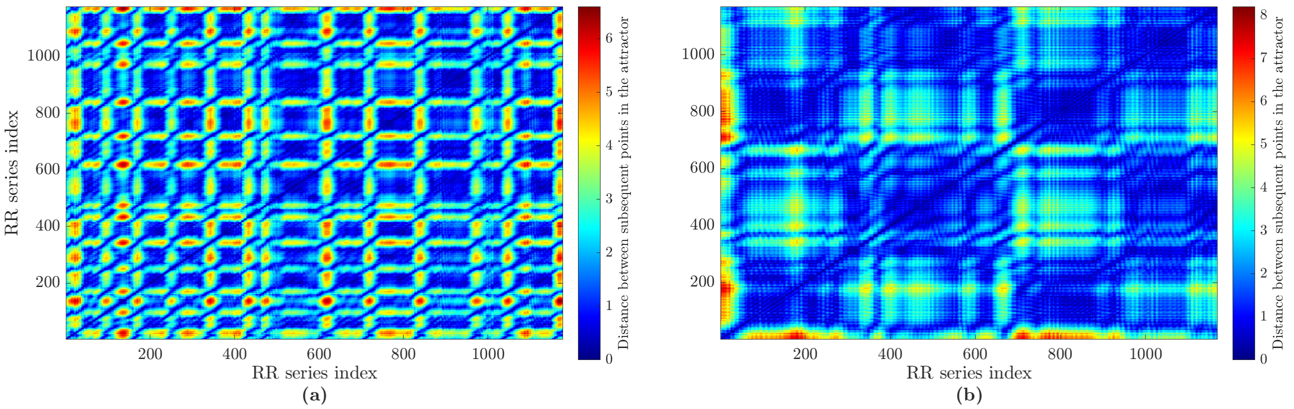

Figure S7: Examples of colour recurrence plots. These were computed for two 5-min windows ranging from (a) 78 to 73 minutes and (b) 26 to 21 minutes before seizure onset, respectively, for the first seizure of patient 2.

- Length of the longest diagonal line (L_{max}).
- Laminarity (LAM).
- Trapping time (TT).
- Shannon entropy (ENT).

Table S2: Extended information on HRV-derived features.

**Linearity/ Domain**	**Features**	**Units**	**ML (s)**	**MVs (%)**	**Characterization**	**Computational Time (s)**
Linear/ Time domain	NN50	count	150	0.09	STV, PSA[1]	$(1.1 \pm 9.5) \times 10^{-4}$
	pNN50	%	150	0.09	STV, PSA[1]	
	SDNN	ms	300	0.16	Overall variance[1,13,14]	
	RMSSD	ms	150	0.09	STV, PSA[1,13,14]	
	SDSD	ms	150	0.09	STV, PSA[1]	
	RRMean	ms	150	0.09		
	RRMin	ms	150	0.09		
	RRMax	ms	150	0.09		
	RRVar	ms	150	0.09		
Linear/ Frequency domain	Total power	ms^2	300	0.16	Overall variance[1,13,14]	0.03 ± 0.03
	VLF power	ms^2	300	0.16	LTV[21]	
	LF power	ms^2	150	0.09	SA[14,21]	
	HF power	ms^2	150	0.09	STV, PSA[21]	
	LF/HF	–	150	0.09	SVB[21]	
	LF norm	n.u.	150	0.09	SA	
	HF norm	n.u.	150	0.09	STV, PSA	
Nonlinear	SD_1	ms	150	0.09	STV, PSA[9,23]	$(4.8 \pm 40.4) \times 10^{-5}$
	SD_2	ms	150	0.09	Overall variance[9,23]	
	SD_1/SD_2	–	150	0.09	SVB[9]	
	DFA α_1	–	300	0.16	STV[23]	$(4.4 \pm 1.6) \times 10^{-3}$
	DFA α_2	–	300	0.16	LTV, SA[23]	
	ApEn	–	180	0.10	STV, PSA[23]	0.03 ± 0.02
	SampEn	–	150	0.09	SVB[23]	$(1.2 \pm 0.5) \times 10^{-3}$
	LLE	–	300	0.16		0.8 ± 0.2
	CD	–	300	0.16		2.1 ± 12.5
	RQA (REC, L, TT, DET, LAM, ENT, L_{max})	–	150	0.09		1.3 ± 3.6

s: seconds; ms: milli seconds; n.u.: normalized units; ML: minimum length of clean ECG recordings required to compute each feature[1,9]; MVs: percentage ratio of the number of missing values (i.e., 5-min windows contaminated with noise and therefore not having the ML required) to the total of 658,784 5-min windows (238 seizures times 2,768 5-min windows) analysed for each feature. Each feature can be characterized in terms of (i) ANS branch activation: sympatho-vagal balance (or sympathetic-parasympathetic), SVB; sympathetic activity, SA; and parasympathetic activity, PSA; and (ii) short-term variability, STV and long-term variability, LTV. Computational time (averaged over all 5-min windows) is presented as the average ± standard deviation. All calculations were performed in MATLAB R2019b, running on WINDOWS 10 Pro with an INTEL Core i7-4790K CPU at 4 GHz, and 32 GB RAM.

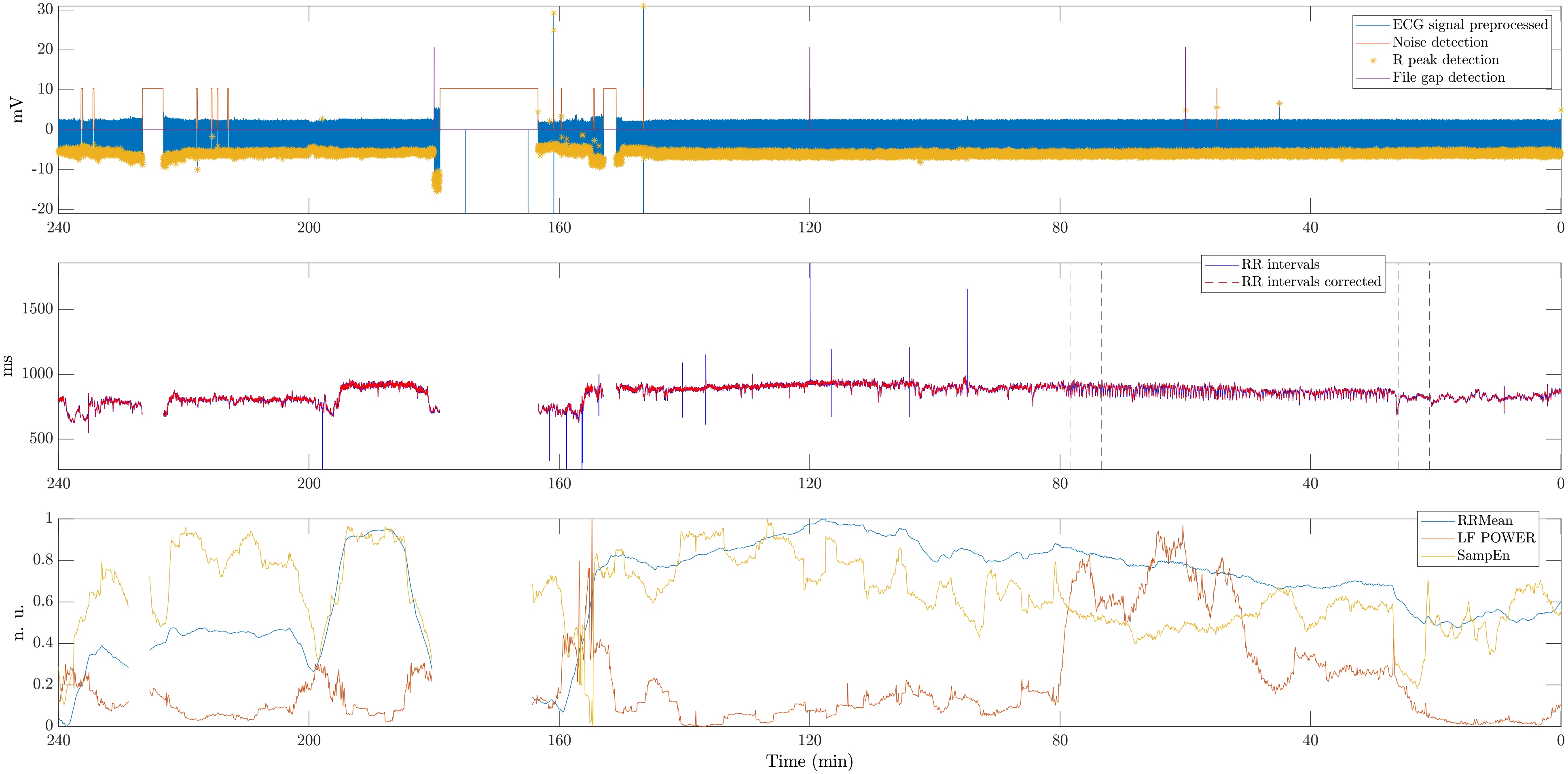

Figure S8: Example of the results returned for: (i) preprocessing and noise detection (top plot); (ii) RRIs series editing (middle plot); and (iii) HRV feature engineering (bottom plot) steps, for the first seizure of patient 2 (identified by 8902 in Table S1). The feature combination $c = 2159$, corresponding to features RRMean, LF Power and SampEn, is depicted. Seizure onset occurs at 0 min, as the x-axis represents the time anticipating seizure onset. The vertical dashed lines indicate the location of the 5-min windows depicted in Figures S5, S6 and S7.

5 Searching for preictal patterns using unsupervised learning

Algorithm 1 Selecting clustering solutions

1. Initialize accepted solutions set $A = []$;
2. For $c = 1$ to C:
   (a) Set condition 1: clustering solution comprising two clusters.
   (b) Set condition 2: clustering solution evaluated with $DI >= 0.15$.
   (c) Set condition 3: clustering solution not containing noisy samples.
   (d) Set condition 4: clustering solution containing smaller cluster with 20 or more samples.
   (e) If (condition 1 & condition 2 & condition 3 & condition 4)
      i. Accept clustering solution stored in A.
   (f) Else
      i. Discard clustering solution.

Algorithm 2 Selecting clustering solutions based on time continuity and duration

1. Initialize accepted solutions $A_1 = []$.
2. Initialize solutions information matrix $M = cell(A, 2)$.
3. For $a = 1$ to A:
   (a) If smaller cluster is continuous over time
      i. $M(a, 1) =$ "*continuous*".
      ii. Find the solution(s) with the smaller cluster comprising the highest number of samples, save it in A_1 and register the number of samples in $M(a, 2)$.
   (b) Else
      i. $M(a, 1) =$ "*discontinuous*".
      ii. Find the solution(s) with the smaller cluster comprising the highest number of samples, save it in A_1 and register the number of samples in $M(a, 2)$.

Algorithm 3 Stratifying and selecting clustering solutions based on time continuity and duration

1. Initialize accepted solutions sets for each of the intervals under analysis.
   $A_{[40,0]} = []$
   $A_{[80,40]} = []$
   $A_{[120,80]} = []$
2. For $a = 1$ to A:
   (a) If smaller cluster located in [40, 0] interval before seizure
      i. Save solution in $A_{[40,0]}$.
   (b) Elseif smaller cluster located in [80, 40] interval before seizure
      i. Save solution in $A_{[80,40]}$.
   (c) Elseif smaller cluster located in [120, 80] interval before seizure
      i. Save solution in $A_{[120,80]}$.
   (d) Else
      i. Discard clustering solution.
   (e) Select solutions in $A_{[40,0]}$ according to Algorithm 2.
   (f) Select solutions in $A_{[80,40]}$ according to Algorithm 2.
   (g) Select solutions in $A_{[120,80]}$ according to Algorithm 2.

References

1. Electrophysiology, T. F. o. t. E. S. o. C., American, T. N., & Electrophysiology, S. o. P. Heart Rate Variability. *Circulation* **93**, 1043–1065, DOI: 10.1161/01.CIR.93.5.1043 (1996). WOS:A1996UF54400011.
2. Clifford, G. D. Linear Filtering Methods. In Clifford, G. D., Azuaje, F. & Mcsharry, P. E. (eds.) *Advanced Methods and Tools for ECG Data Analysis*, chap. 5, 135–170 (Artech House, 2006).
3. Sörnmo, L. & Laguna, P. ECG Signal Processing: Heart Rate Variability. In Sörnmo, L. & Laguna, P. (eds.) *Bioelectrical Signal Processing in Cardiac and Neurological Applications*, chap. 8, 567–631, DOI: 10.1016/B978-012437552-9/50008-8 (Elsevier, 2005), 1st edn.
4. Clifford, G. D. Introduction to Feature Extraction. In Clifford, G. D., Azuaje, F. & Mcsharry, P. E. (eds.) *Advanced Methods and Tools for ECG Data Analysis*, chap. 9, 245–268 (Artech House, 2006).
5. Sörnmo, L. & Laguna, P. ECG Signal Processing. In Sörnmo, L. & Laguna, P. (eds.) *Bioelectrical Signal Processing in Cardiac and Neurological Applications*, chap. 7, 453–566, DOI: 10.1016/B978-012437552-9/50007-6 (Elsevier, 2005), 1st edn.
6. Pan, J. & Tompkins, W. J. A Real-Time QRS Detection Algorithm. *IEEE Transactions on Biomed. Eng.* **BME-32**, 230–236, DOI: 10.1109/TBME.1985.325532 (1985).
7. Clifford, G. D. ECG Statistics, Noise, Artifacts, and Missing Data. In Clifford, G. D., Azuaje, F. & Mcsharry, P. E. (eds.) *Advanced Methods and Tools for ECG Data Analysis*, chap. 3, 55–100 (Artech House, 2006).
8. Peltola, M. A. Role of editing of R–R intervals in the analysis of heart rate variability. *Front. Physiol.* **3**, 1–10, DOI: 10.3389/fphys.2012.00148 (2012).
9. Shaffer, F. & Ginsberg, J. P. An Overview of Heart Rate Variability Metrics and Norms. *Front. Public Heal.* **5**, 1–17, DOI: 10.3389/fpubh.2017.00258 (2017).
10. Jansen, K. & Lagae, L. Cardiac changes in epilepsy. *Seizure* **19**, 455–460, DOI: 10.1016/j.seizure.2010.07.008 (2010).
11. Devinsky, O. Effects of Seizures on Autonomic and Cardiovascular Function. *Epilepsy Curr.* **4**, 43–46, DOI: 10.1111/j.1535-7597.2004.42001.x (2004).
12. Delamont, R. S. & Walker, M. C. Pre-ictal autonomic changes. *Epilepsy Res.* **97**, 267–272, DOI: 10.1016/j.eplepsyres.2011.10.016 (2011).
13. van der Kruijs, S. J. *et al.* Autonomic nervous system functioning associated with psychogenic nonepileptic seizures: Analysis of heart rate variability. *Epilepsy & Behav.* **54**, 14–19, DOI: 10.1016/j.yebeh.2015.10.014 (2016).
14. Ponnusamy, A., Marques, J. L. & Reuber, M. Comparison of heart rate variability parameters during complex partial seizures and psychogenic nonepileptic seizures. *Epilepsia* **53**, 1314–1321, DOI: 10.1111/j.1528-1167.2012.03518.x (2012).
15. VanderPlas, J. T. Understanding the Lomb–Scargle Periodogram. *The Astrophys. J. Suppl. Ser.* **236**, 1–28, DOI: 10.3847/1538-4365/aab766 (2018). 1703.09824.
16. de Zambotti, M., Trinder, J., Silvani, A., Colrain, I. M. & Baker, F. C. Dynamic coupling between the central and autonomic nervous systems during sleep: A review. *Neurosci. & Biobehav. Rev.* **90**, 84–103, DOI: 10.1016/j.neubiorev.2018.03.027 (2018).
17. Bartels, R., Neumamm, L., Peçanha, T. & Carvalho, A. R. S. SinusCor: an advanced tool for heart rate variability analysis. *BioMedical Eng. OnLine* **16**, 1–15, DOI: 10.1186/s12938-017-0401-4 (2017).
18. Billman, G. E. Heart Rate Variability ? A Historical Perspective. *Front. Physiol.* **2**, 1–13, DOI: 10.3389/fphys.2011.00086 (2011).
19. Kuusela, T. Methodological Aspects of Heart Rate Variability Analysis. In Kamath, M. V., Watanabe, M. A. & Upton, A. R. M. (eds.) *Heart Rate Variability (HRV) Signal Analysis*, chap. 2, 9–42 (CRC Press, 2012), 1st edn.
20. Tarvainen, M. P., Niskanen, J.-P., Lipponen, J. A., Ranta-aho, P. O. & Karjalainen, P. A. Kubios HRV – Heart rate variability analysis software. *Comput. Methods Programs Biomed.* **113**, 210–220, DOI: 10.1016/j.cmpb.2013.07.024 (2014).
21. Behbahani, S. A Review of Significant Research on Epileptic Seizure Detection and Prediction using Heart Rate Variability. *Turk Kardiyoloji Dernegi Arsivi - Arch. Turkish Soc. Cardiol.* **46**, 414–421, DOI: 10.5543/tkda.2018.64928 (2018).

22. Lotufo, P. A., Valiengo, L., Benseñor, I. M. & Brunoni, A. R. A systematic review and meta-analysis of heart rate variability in epilepsy and antiepileptic drugs. *Epilepsia* **53**, 272–282, DOI: 10.1111/j.1528-1167.2011.03361.x (2012).

23. Voss, A., Schulz, S., Schroeder, R., Baumert, M. & Caminal, P. Methods derived from nonlinear dynamics for analysing heart rate variability. *Philos. Transactions Royal Soc. A: Math. Phys. Eng. Sci.* **367**, 277–296, DOI: 10.1098/rsta.2008.0232 (2009).

24. Maestri, R. *et al.* Assessing nonlinear properties of heart rate variability from short-term recordings: are these measurements reliable? *Physiol. Meas.* **28**, 1067–1077, DOI: 10.1088/0967-3334/28/9/008 (2007).

25. Hoshi, R. A., Pastre, C. M., Vanderlei, L. C. M. & Godoy, M. F. Poincaré plot indexes of heart rate variability: Relationships with other nonlinear variables. *Auton. Neurosci.* **177**, 271–274, DOI: 10.1016/j.autneu.2013.05.004 (2013).

26. Peng, C., Havlin, S., Stanley, H. E. & Goldberger, A. L. Quantification of scaling exponents and crossover phenomena in nonstationary heartbeat time series. *Chaos: An Interdiscip. J. Nonlinear Sci.* **5**, 82–87, DOI: 10.1063/1.166141 (1995). arXiv:1011.1669v3.

27. Costa, M. D., Davis, R. B. & Goldberger, A. L. Heart Rate Fragmentation: A New Approach to the Analysis of Cardiac Interbeat Interval Dynamics. *Front. Physiol.* **8**, 1–13, DOI: 10.3389/fphys.2017.00255 (2017).

28. Pincus, S. M. Approximate entropy as a measure of system complexity. *Proc. Natl. Acad. Sci.* **88**, 2297–2301, DOI: 10.1073/pnas.88.6.2297 (1991).

29. Richman, J. S. & Moorman, J. R. Physiological time-series analysis using approximate entropy and sample entropy. *Am. J. Physiol. Circ. Physiol.* **278**, H2039–H2049, DOI: 10.1152/ajpheart.2000.278.6.H2039 (2000).

30. Delgado-Bonal, A. & Marshak, A. Approximate Entropy and Sample Entropy: A Comprehensive Tutorial. *Entropy* **21**, 1–37, DOI: 10.3390/e21060541 (2019).

31. Varsavsky, A., Mareels, I. & Cook, M. *Epileptic seizure and the EEG: Measurement, Models, Detection and Prediction* (CRC Press, Boca Raton, FL, 2011).

32. Rajendra Acharya, U., Paul Joseph, K., Kannathal, N., Lim, C. M. & Suri, J. S. Heart rate variability: a review. *Med. & Biol. Eng. & Comput.* **44**, 1031–1051, DOI: 10.1007/s11517-006-0119-0 (2006). s11517-006-0119-0.

33. Henriques, T. *et al.* Nonlinear Methods Most Applied to Heart-Rate Time Series: A Review. *Entropy* **22**, 1–39, DOI: 10.3390/e22030309 (2020).

34. Lekscha, J. & Donner, R. V. Phase space reconstruction for non-uniformly sampled noisy time series. *Chaos: An Interdiscip. J. Nonlinear Sci.* **28**, 1–12, DOI: 10.1063/1.5023860 (2018).

35. Kennel, M. B., Brown, R. & Abarbanel, H. D. I. Determining embedding dimension for phase-space reconstruction using a geometrical construction. *Phys. Rev. A* **45**, 3403–3411, DOI: 10.1103/PhysRevA.45.3403 (1992). arXiv:1011.1669v3.

36. Fraser, A. M. & Swinney, H. L. Independent coordinates for strange attractors from mutual information. *Phys. Rev. A* **33**, 1134–1140, DOI: 10.1103/PhysRevA.33.1134 (1986).

37. Rosenstein, M. T., Collins, J. J. & De Luca, C. J. A practical method for calculating largest Lyapunov exponents from small data sets. *Phys. D: Nonlinear Phenom.* **65**, 117–134, DOI: 10.1016/0167-2789(93)90009-P (1993).

38. Grassberger, P. & Procaccia, I. Characterization of Strange Attractors. *Phys. Rev. Lett.* **50**, 346–349, DOI: 10.1103/PhysRevLett.50.346 (1983).

39. Eckmann, J.-P., Kamphorst, S. O. & Ruelle, D. Recurrence Plots of Dynamical Systems. *Europhys. Lett. (EPL)* **4**, 973–977, DOI: 10.1209/0295-5075/4/9/004 (1987).

40. Billeci, L., Marino, D., Insana, L., Vatti, G. & Varanini, M. Patient-specific seizure prediction based on heart rate variability and recurrence quantification analysis. *PLOS ONE* **13**, 1–21, DOI: 10.1371/journal.pone.0204339 (2018).

41. MARWAN, N., CARMENROMANO, M., THIEL, M. & KURTHS, J. Recurrence plots for the analysis of complex systems. *Phys. Reports* **438**, 237–329, DOI: 10.1016/j.physrep.2006.11.001 (2007).

42. Marwan, N. & Webber, C. L. Mathematical and Computational Foundations of Recurrence Quantifications. In Webber, J. C. & Marwan, N. (eds.) *Recurrence Quantification Analysis. Understanding Complex Systems.*, chap. 1, 3–43, DOI: 10.1007/978-3-319-07155-8_1 (Springer, Cham, 2015).
